# Advancing surgical instrument safety: A screen of oxidative and alkaline prion decontaminants using real-time quaking-induced conversion with prion-coated steel beads as surgical instrument mimetic

**DOI:** 10.1371/journal.pone.0304603

**Published:** 2024-06-13

**Authors:** Daniel Heinzer, Merve Avar, Manuela Pfammatter, Rita Moos, Petra Schwarz, Matthias T. Buhmann, Benjamin Kuhn, Stefan Mauerhofer, Urs Rosenberg, Adriano Aguzzi, Simone Hornemann

**Affiliations:** 1 Institute of Neuropathology, University of Zurich, Zurich, Switzerland; 2 Borer Chemie AG, Zuchwil, Switzerland; The University of Texas Health Science Center at Houston, UNITED STATES

## Abstract

Iatrogenic transmission of prions, the infectious agents of fatal Creutzfeldt-Jakob disease, through inefficiently decontaminated medical instruments remains a critical issue. Harsh chemical treatments are effective, but not suited for routine reprocessing of reusable surgical instruments in medical cleaning and disinfection processes due to material incompatibilities. The identification of mild detergents with activity against prions is therefore of high interest but laborious due to the low throughput of traditional assays measuring prion infectivity. Here, we report the establishment of TESSA (s**T**ainl**ES**s steel-bead **S**eed **A**mplification assay), a modified real-time quaking induced cyclic amplification (RT-QuIC) assay that explores the propagation activity of prions with stainless steel beads. TESSA was applied for the screening of about 70 different commercially available and novel formulations and conditions for their prion inactivation efficacy. One hypochlorite-based formulation, two commercially available alkaline formulations and a manual alkaline pre-cleaner were found to be highly effective in inactivating prions under conditions simulating automated washer-disinfector cleaning processes. The efficacy of these formulations was confirmed *in vivo* in a murine prion infectivity bioassay, yielding a reduction of the prion titer for bead surface adsorbed prions below detectability. Our data suggest that TESSA represents an effective method for a rapid screening of prion-inactivating detergents, and that alkaline and oxidative formulations are promising in reducing the risk of potential iatrogenic prion transmission through insufficiently decontaminated instrument surfaces.

## Introduction

Transmissible spongiform encephalopathies (TSEs), or prion diseases, represent a group of fatal disorders which are hallmarked by the misfolding and aggregation of the cellular prion protein (PrP^C^) into a pathological, proteinase K (PK) resistant form, PrP^Sc^ [1]. The infectious agent of prion diseases, the prion, can occur as different strains which are characterized by different inheritable physicochemical properties, leading to a variety of clinical signs [2]. It is widely accepted that the different strain properties are encoded in the different conformations of the prion agent [3]. Prion diseases can be inherited or arise sporadically as Creutzfeldt-Jakob disease (sCJD) in humans [4]. In addition, the nature of the prion allows manifestation of the disease through exposure to infected tissue via ingestion or through iatrogenic transmission [4]. The first recorded case of iatrogenic CJD (iCJD) as a result of a corneal transplant operation dates back to 1974 [5]. Later reports have shown that prion diseases are transmissible through contact with contaminated surgical devices, dura-mater transplants, and human growth hormone administration [6–8].

The decontamination of prions poses a major challenge, as they are incredibly resistant to common cleaning, disinfection and sterilization processes [9–11] which were originally developed for the inactivation of viruses and microorganisms [12–15]. Moreover, prions strongly adhere to a broad variety of surface materials [15–19], which further complicates their removal and decontamination by conventional cleaning and instrumental reprocessing routines, leaving a potential risk of transmission of prion diseases through insufficiently decontaminated medical devices. The World Health Organization (WHO) has therefore published a recommendation list of strong chemical and physical procedures that should be applied for the decontamination of prions [20]. Such procedures, however, are not applicable to routine, daily medical device reprocessing or to non-disposable medical devices, such as endoscopes and surgical instruments, due to material incompatibilities [21]. Also, preventive measures such as the utilization of single-use tools, or allocating instruments only for the use on potential CJD cases, are not reasonable solutions, as they would lead to insurmountable costs with questionable feasibility [22]. Hence, there is still a need for mild prion decontamination products that can be applied for the reprocessing of surgical instruments.

To date, the efficacy of novel prion decontaminants is routinely assessed in rodent bioassays that report on prion infectivity based on the survival of indicator mice inoculated with prions [23–26]. However, rodent assays are limited in throughput, cost-intensive and take several months to complete. In addition, *in vivo* bioassays should be replaced by *in vitro* assays, where possible, in compliance with the 3R of animal welfare. The recent development of *in vitro* seed amplification assays (SAA), such as the protein misfolding cyclic amplification (PMCA) [27] and real-time quaking induced cyclic amplification assay (RT-QuIC) [28–30] that take advantage of the seeding capabilities of prions allows to conduct several experiments in parallel in a much shorter time with higher throughput. In this study, we report the development of a modified RT-QuIC assay termed TESSA (s**T**ainl**ES**s steel-bead **SA**A), that uses stainless steel beads as prion carriers, to mimic the steel surface of surgical instruments. We applied TESSA for a screen of a series of formulations and conditions to explore their prion-inactivating (prionicidal) capacity for automated medical device reprocessing. A hypochlorite-based formulation was identified as the most efficient prion decontaminant in the screen and was further validated together with two commercially available alkaline formulations and an alkaline pre-cleaner in a mouse bioassay. All products reduced prion titers below detectability in the bioassay, demonstrating the usefulness of these formulations as potential prion decontaminants and TESSA as a valuable tool for the rapid evaluation of novel prionicidals.

## Results

### Establishment of TESSA

**Fig 1:**
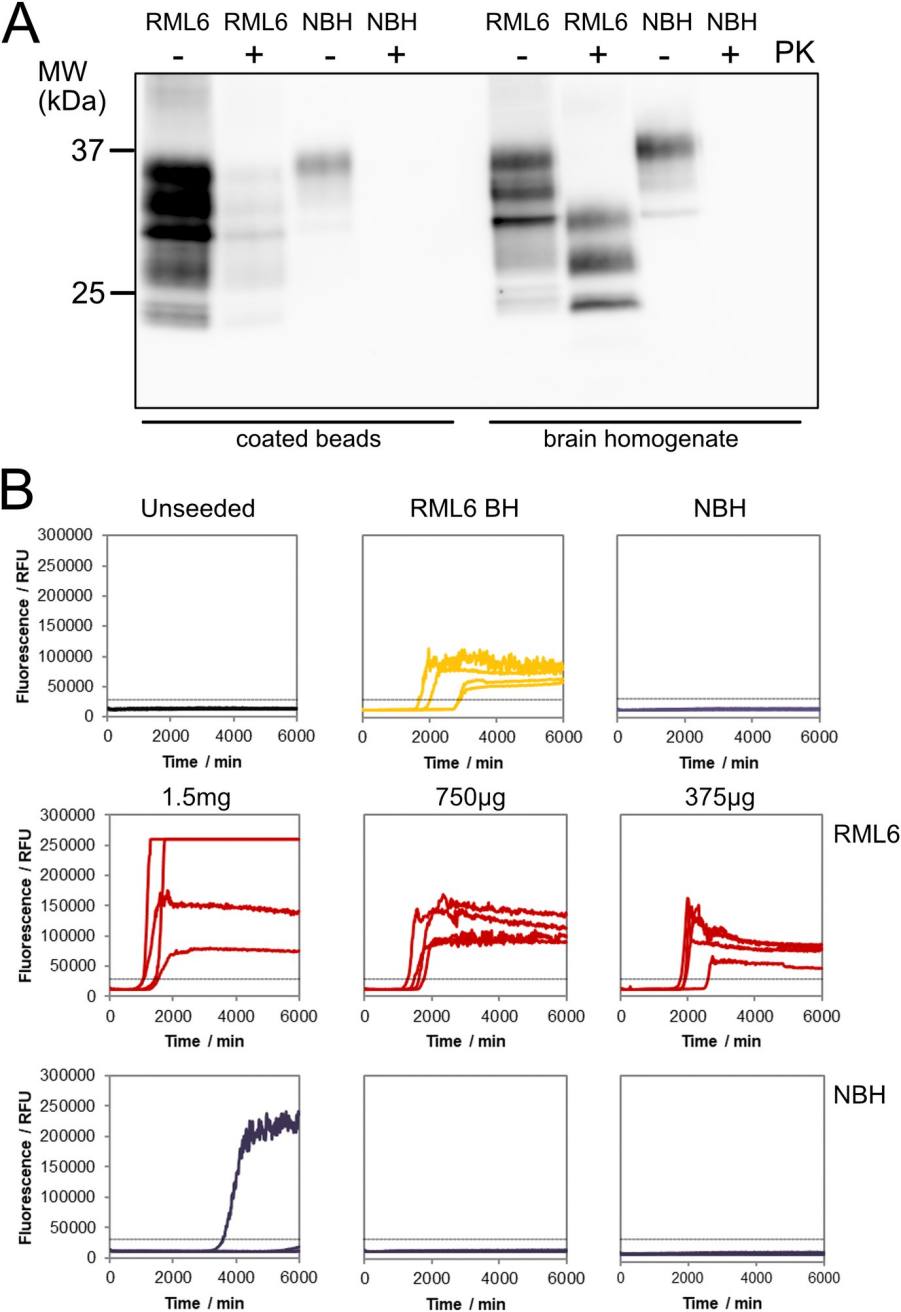
Evaluation of PrP^Sc^-specific binding to the stainless-steel beads. **(A)** PK-immunoblot analysis demonstrating the binding of total PrP and PrP^Sc^ to the beads. NBH- and RML6-exposed beads were blotted after treating the samples without (-) or with (+) PK for the detection of total PrP and PrP^Sc^. As controls RML6 and NBH without and with PK digestion were loaded. The in-house produced anti-PrP antibody POM-1 [36] was used for detection. The molecular weight standard (MW) is shown in kilodaltons (kDa). **(B)** TESSA analysis showing specific propagation activity for three different amounts of RML6-coated beads as indicated in the Figure. NBH-coated beads, unseeded reactions, NBH and RML6 brain homogenate (RML6 BH) were used as negative and positive controls, respectively. All reactions were performed in quadruplicates. The dashed line indicates the ThT fluorescence threshold for a positive reaction.

To develop an assay that allows for the fast screening of novel anti-prion decontaminants, we modified the RT-QuIC assay [28, 31] for the detection of surface-attached prions using micron-sized stainless-steel beads as prion carriers (AISI 316L). The prion-exposed steel beads were first tested for their capability to efficiently bind prions. Beads were exposed to either prion-infected mouse brain homogenate (1% (w/v) RML6, Rocky Mountain Laboratory strain, passage 6 [32]) or non-infectious brain homogenate (NBH) and analyzed by immunoblotting for surface bound PrP^C^ and proteinase K (PK)-resistant PrP^Sc^, as a surrogate marker for the presence of prions [33]. Total PrP was detectable on non-digested RML6- and NBH-coated beads (Fig 1A), confirming efficient binding of PrP to the steel beads. After PK digestion, PrP^C^ was completely digested in the NBH reference sample and on NBH-coated beads, whereas the RML6 sample displayed the typical electrophoretic mobility pattern of the PK-resistant core fragments of PrP^Sc^ [34, 35]. PK-digested RML6-coated beads, however, showed a decreased signal intensity and no changes in the electrophoretic distribution pattern when compared to the undigested beads. This finding suggests that the PK cleavage sites of PrP^Sc^ could have become protected due to the adsorption of PrP^Sc^ to the beads, so that PrP^Sc^ largely remained preserved as full-length protein on the beads.

To further assess whether prions, upon adhering to the beads, retained their characteristic propagation activity and to identify the maximum amount of beads with the highest specific sensitivity in the TESSA, we analyzed the seeding properties of three different amounts of beads coated with either RML6 or NBH (1.5 mg, 750 μg and 375 μg/TESSA reaction, Fig 1B). Efficient seeding was detected for all amplification reactions of RML6-coated beads (Fig 1B). These data demonstrate that prions retained their propagation activity after adsorption to the beads. For the NBH-coated beads, one non-specific positive reaction out of four replicates was detected at the highest amount of 1.5 mg beads/TESSA reaction. To avoid any potential risk of non-specific positive reactions and technical difficulties in the handling of the highest bead amount due to the high viscosity of the bead suspension, we chose 750 μg beads per TESSA reaction as the optimal amount for the formulation screening (for TESSA raw data see S1 Dataset).

### Applicability of TESSA as a method for testing prion decontaminants

To investigate the applicability of TESSA for the assessment of new prion decontaminants, we first evaluated its performance with prion-exposed beads treated with 1 M NaOH (2 h, RT), a common standard decontamination method for the inactivation of prions [20], and three commercially available alkaline formulations. These formulations included (Table 1): (i) deconex^®^ 28 ALKA ONE-x (28AO); an alkaline cleaner whose alkalinity is solely based on potassium metasilicate and that has been shown to be effective for the decontamination of the Chandler strain of prion diseases *in vitro* [37], (ii) 28AO in combination with deconex^®^ TWIN ZYME (28AO/TZ); a mild alkaline product with improved cleansing properties through the enzymatic activities of subtilisin and α-amylase, and (iii) deconex^®^ 36 BS ALKA (36BS); a non-enzymatic highly alkaline pre-cleaner that was presumed to exhibit its prion decontamination efficacy due to a pH-value of >11, though still being applicable to a broad range of materials despite its relatively high pH-value.

**Table 1:** Chemical composition and conditions of the alkaline formulations used in the applicability testing of TESSA.

Formulation	Ingredients	Experimental conditions	TESSA reactions (positive/total) ^§^
28AO	Tripotassium orthophosphate (15–30%) Dipotassium Trioxosilicate (5–15%) Amphoteric surfactants (< 5%)	Concentration: 1% in ddH_2_O Condition: 70°C, 10 min	4/16
28AO/TZ	• N,N-Dimethyldecylamine N-oxide (1–5%) • Subtilisin (< 1%) • Alpha-Amylase (< 1%) • Non-ionic surfactants (15–30%) • Potassium sorbate (5%)	Concentration: 1% 28AO + 0.3% TZ in ddH_2_O Two step reprocessing 1. 45°C, 10 min 2. 70°C, 10 min	0/16
36BS	Tripotassium orthophosphate (15–30%) Anionic surfactants (< 5%) Non-ionic surfactants (< 5%) Alanine, N,N-bis(carboxymethyl) sodium salt (5–15%)	Concentration: 2% in water with standardized hardness Condition: 25°C, 60 min	2/16

^§^ Graphs are presented in Fig 2A and S1 Fig.

**Fig 2:**
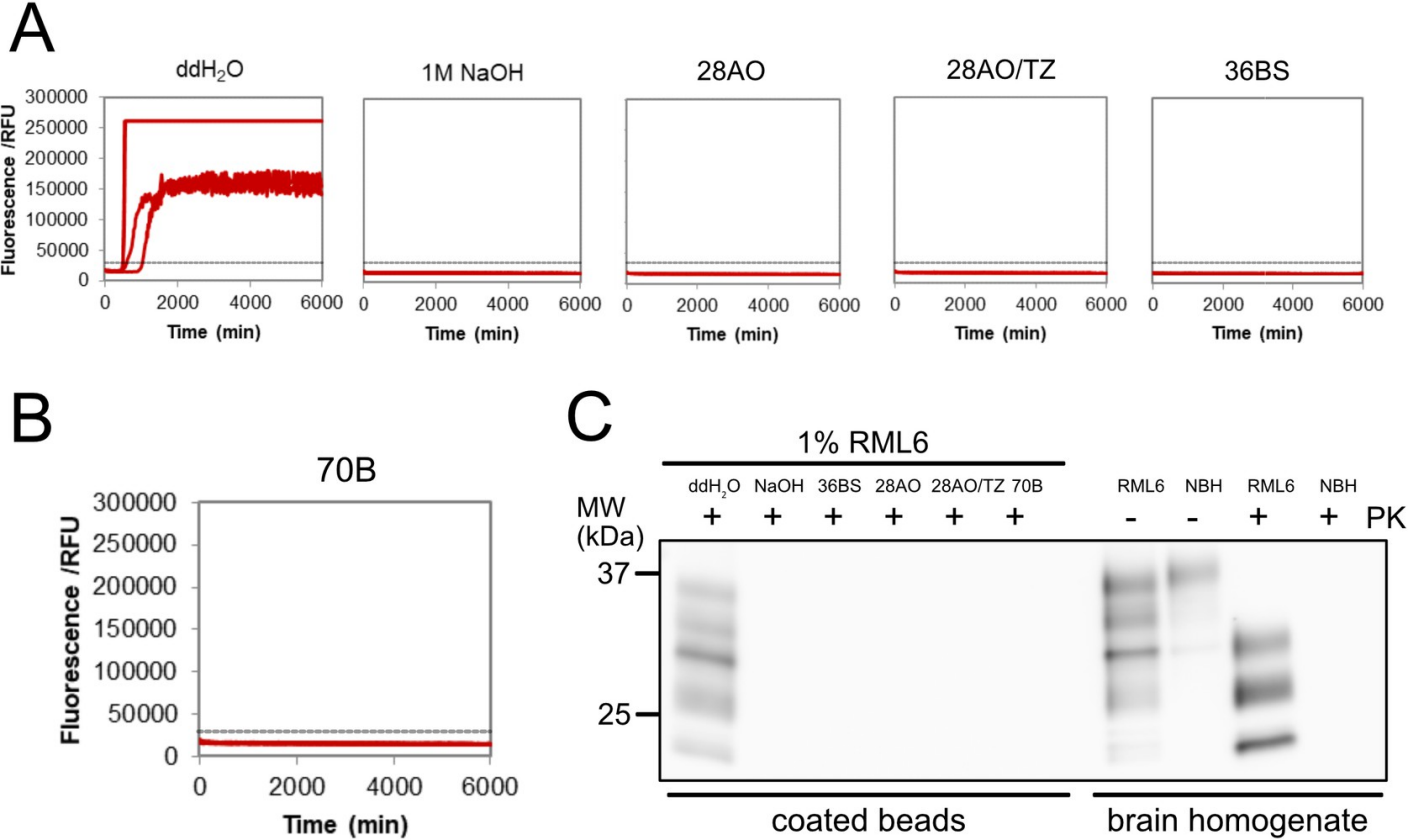
TESSA and PK-immunoblot analysis to assess the decontamination effectiveness of the four lead formulations. **(A)** TESSA analysis of RML6-exposed and treated beads. The decontamination of RML6-exposed beads with the different formulations efficiently abolished the seeding activity in TESSA. RML6-exposed beads treated with ddH_2_O and NaOH were used as positive and negative controls, respectively. Shown is one representative data set with quadruplicate TESSA reactions for each condition (see also S1 and S2 Figs). **(B)** Same as (A), but after decontamination with 70B. **(C)** PK-immunoblot analysis of RML6-exposed and treated beads. The decontamination of RML6-exposed beads with the different formulations efficiently abolished the PK-immunoblot signal. RML6-exposed beads treated with ddH_2_O and NaOH as well as RML6 and NBH before (-) and after PK (+) digestion are shown as controls.

Beads were exposed to RML6 (1% (w/v)) and treated with either NaOH (2 h, RT) or the three different formulations 28AO, 28AO/TZ or BS under conditions recommended by the manufacturer (Table 1) and analyzed by TESSA. Prion-exposed beads treated with deionized water (ddH_2_O) maintained their prion seeding characteristics, while those treated with NaOH used as a control did not elicit any response in TESSA (Fig 2A and S2 Fig). Additionally, none of the 16 TESSA reactions for RML6-coated beads treated with 28AO/TZ exhibited a detectable response (Fig 2A and S1 Fig). In the case of 36BS and 28AO (S1A Fig), 2 and 4 out of 16 reactions, respectively, showed a slightly positive response near our determined threshold (~30,000 RFU) and cut-off time (~5,000 min) for positive reactions. Given their close proximity to the threshold and delayed occurrence within the TESSA run, these reactions can be considered as errors false-positive reactions, likely resulting from rare spontaneous aggregation events [29]. Therefore, we conclude that all three formulations effectively eliminate the seeding activity of the prion-exposed beads and exhibit promising anti-prion inactivation activities. These data also indicate that TESSA is a suitable *in vitro* tool for the screening and evaluation of the effectiveness of novel prion decontaminants.

### Screening of different formulations for their prion inactivation effectiveness using TESSA

We then applied TESSA to screen about 70 different alkaline and oxidative formulations and conditions for their anti-prion efficacy under typical automated reprocessing conditions used in washer-disinfectors (summarized in Table 2). Based on their main active anti-prion ingredient, the different formulations were grouped into 4 different categories. The first category comprised formulations for which the anti-prion effect was expected to be mediated by the hydrolytic activity of alkanolamines and complexing agents, which are common donors of alkalinity in detergent solutions with a typical pK_a_ of around 9.5. The second category included alkaline enzymatic phosphate/silicate-based formulations (TWIN PH10). The alkalinity of these products is based on the phosphate/silicate composition with the advantage of higher material compatibility. Category 3 encompassed a selection of commercially available strong alkaline hydroxide- and hydroxide/silicate-based formulations and solutions, whereas formulations of category 4 were composed of hypochlorite (70B); a chemical that acts as a strong oxidizing reagent under alkaline conditions [38]. To support the anti-prion activity and cleaning properties of the main components, products of categories 1 and 2 were formulated with a series of enzymatic, non-ionic surfactants and surface-active reagents (Table 2).

**Table 2:** Formulations and experimental conditions.

**Category 1: Enzymatic alkanolamine surfactant-based formulations**					
**Formulation**	Composition^#^ (v/v), condition		TESSA reactions (positive/total)*		
**F1, F2**	20D / 10U,10V		4/4*		
**F3-F5**	20D / 11T-11V		4/4*		
**F6-F9**	20D / 40B-40E		4/4*		
**F10, F11**	20H / 10U, 10V		4/4*		
**F12**	20H / 11T		8/16		
**F13**	20H / 11U		7/12		
**F14**	20H / 11V		2/8		
**F15-17**	20H / 40B-40D		4/4*		
**F18**	20H / 40E		4/12		
**F26**	20H / 12A		7/20		
**F27**	20H / 12B		5/8		
**F28-F30**	20H / 12C-12E		3/4*		
**F32**	20K / 12F		6/8		
**F38-F50**	20K / 12K-12W		4/4*		
**F51-F56**	20K / 13A-13F		4/4*		
**F57**	20K / 13G		8/8		
**F58**	20K / 13G, 20 min, 55°C		8/8		
**F59**	0.4% 20K / 0.6% 13G		7/8		
**F60**	0.4% 20K / 0.6% 13G; 20 min, 55°C		4/8		
**F61**	0.4% 20K / 0.6% 13G; 20 min, 65°C		6/8		
*: All formulations in this row yielded identical results.					
20D: Alkanolamine 30–50% (complexing agents 1–5%, amines > 30%, phosphonates <1%)					
20H: Alkanolamine 30–50% (complexing agents 5–10%, amines >30%, phosphonates <1%)					
20K: Alkanolamine 5–15% (complexing agents 5–15%, amines 5–15%, phosphates 15–30%, phosphonates <1%)					
10U-V: enzymatic product with different concentrations of Ca- and Mg-salts, enzymatic components 1–5%					
non-ionic surfactant 1–5%					
11T-V: various enzymatic products, non-ionic surfactants, different concentrations of fatty alcohol alcoxylates 5–10%, enzymatic components <5% and alkylglucoside, glycol 1–5%					
12A-W: 11T, various concentrations of ingredients					
13A-G: various enzymatic products, non-ionic surfactant					
40B-E: 11T-U, lower enzyme concentrations, higher glycol concentration, non-ionic surfactants					
^#^ Formulations F1-F58 are composed of 0.3% (v/v) 20D-20K and 0.2% (v/v) 10U, 10V, 11T-11V, 40B-40E, 12A-13W or 13A-13G					
**Category 2: Enzymatic surfactant phosphate/silicate-based formulations**					
**Formulation**		Composition^#^, condition			TESSA reactions (positive/total)
**F19**		deconex^®^ TWIN PH10 / 11T			5/16
**F20**		deconex^®^ TWIN PH10 / 11V			2/4
**F21**		deconex^®^ TWIN PH10 / 12A			2/8
**F22**		deconex^®^ TWIN PH10 / 12B			2/8
**F23**		deconex^®^ TWIN PH10 / 12C			4/8
**F24**		deconex^®^ TWIN PH10 / 12D			4/8
**F25**		deconex^®^ TWIN PH10 / 12E			3/4
**F31**		deconex^®^ TWIN PH10 / 12F			4/8
11T-11V: various enzymatic products, non-ionic surfactants, different concentrations of fatty alcohol alcoxylates 5–10%, enzymatic components <5% and alkylglucoside, glycol 1–5%					
12A-F: 11T, various concentrations of ingredients					
^#^Formulations are composed of 0.3% (v/v) Dec TWIN PH10: Phosphate/Silicate and 0.2% 11T, 11V or 12A-12F					
**Category 3: Hydroxide-based products and solutions**					
**Formulation**		Composition (v/v), condition	TESSA reactions (positive/total)		
**F67**		1% deconex^®^ OP 152, 40 min	8/16		
**F68**		1% deconex^®^ HT 1501,40 min	0/16		
**F69**		1% deconex^®^ MT 14, 40 min	0/16		
**F74**		0.5% deconex^®^ MT 14	3/8		
**F72/73**		1% deconex^®^ MT 14	4/24		
**F75**		2% deconex^®^ MT 14	4/8		
**F76**		3% deconex^®^ MT 14	3/8		
**Category 4: Hypochlorite-based formulations**					
**Formulation**		Composition (v/v)^#^, condition		TESSA reactions (positive/total)	
**F65**		0.5% 70B, 0.5% enzyme and surfactant, 40 min			1/8
**F66**		1% 70B, 40 min			0/16
**F71**		1% 70B			9/24
F71.2^§^		1% 70B, freshly prepared			0/16
**F77**		0.3% 70B			4/20
**F77.2**		0.3% 70B, 5 min, freshly prepared			2/8
**F78**		0.5% 70B			4/20
**F78.2**		0.5% 70B, 5 min, freshly prepared			0/8
**F81**		0.75% 70B			3/8
70B: Tetrapotassium pyrophosphate (15–30%), dipotassium trioxosilicate (5–15%), sodium hypochlorite solution (active chlorine (< 5%))					
^§^ Graphs are presented in Fig 2B and S2B Fig.					

Post-hoc classification of the formulations and experimental conditions. The number of positive replicates from up to 5 independent TESSA experiments with 4 replicates each are shown. All formulations were applied under machine reprocessing conditions (55°C, 10 min), if not otherwise indicated.

We primarily tested the effectiveness of the different formulations under typical automated reprocessing conditions used in washer-disinfectors (10 min, 55°C). Some formulations (F58, F60, F61 and F65-F69) were also applied for longer treatment times (20 and 40 min, respectively, see Table 2) and at further temperatures to assess their overall capability to inactivate the seeding activity of the prion-coated beads. Beads were treated as described above and analyzed by TESSA with the number of replicates as specified in Table 2. Negative and positive controls were included on each microplate, using beads treated with ddH_2_O and NaOH, respectively (see S2A Fig). Formulations were deemed effective when the positivity rate remained at 25% or below, which takes the rare occurrence of false-positive reactions into account, and additional TESSA replicates were performed for these formulations.

The initial assessment of formulations of categories 1 and 2 involved testing them in their standard compositions, with subsequent evaluations conducted using further compositions or other concentrations (Table 2), whereas formulations of categories 3 and 4 were mostly tested at different concentrations. While some formulations reduced the prion seeding activity to undetectable levels during extended treatment times (20 and 40 min), the majority exhibited only moderate to no inactivation efficiency when applied at 55°C for 10 min. Among the tested formulations, F14, F21, F22 and F72/F73 were identified as similarly effective as our initial reference formulations. However, when tested at other conditions or concentrations (Table 2), they proved to be less effective and were consequently excluded from further evaluation.

The most effective formulation under the conditions tested, however, was the hypochlorite-based formulation 70B. Although the data for 70B exhibited some variability over time, attributed to hypochlorite degradation due to the limited shelf life of chlorine-containing products [39], when prepared freshly, 70B eliminated all detectable prion seeding activity (Fig 2B, S2B Fig and Table 2). Formulation 70B also demonstrated effectiveness at an even lower concentration and shorter contact time of 0.5% (v/v) and 5 min (Table 2). To ensure that the efficacy of 70B was not attributed to its auxiliary ingredients, we also tested the single components and raw products that were part of formulation 70B (S1 Table). None of these ingredients showed any effect on the seeding efficacy, demonstrating that the individual ingredients have no prion inactivation properties. Additionally, NBH-exposed beads treated with either 1% or 2% 70B as further control did not induce a signal in TESSA (S1 Table). Our results thus indicate that formulation 70B had the highest prion decontamination capability among the tested products in the screen, but with the limitation of the stability of hypochlorite containing formulations [9, 39, 40].

### Efficacy confirmation by immunoblotting and in a mouse bioassay

**Fig 3:**
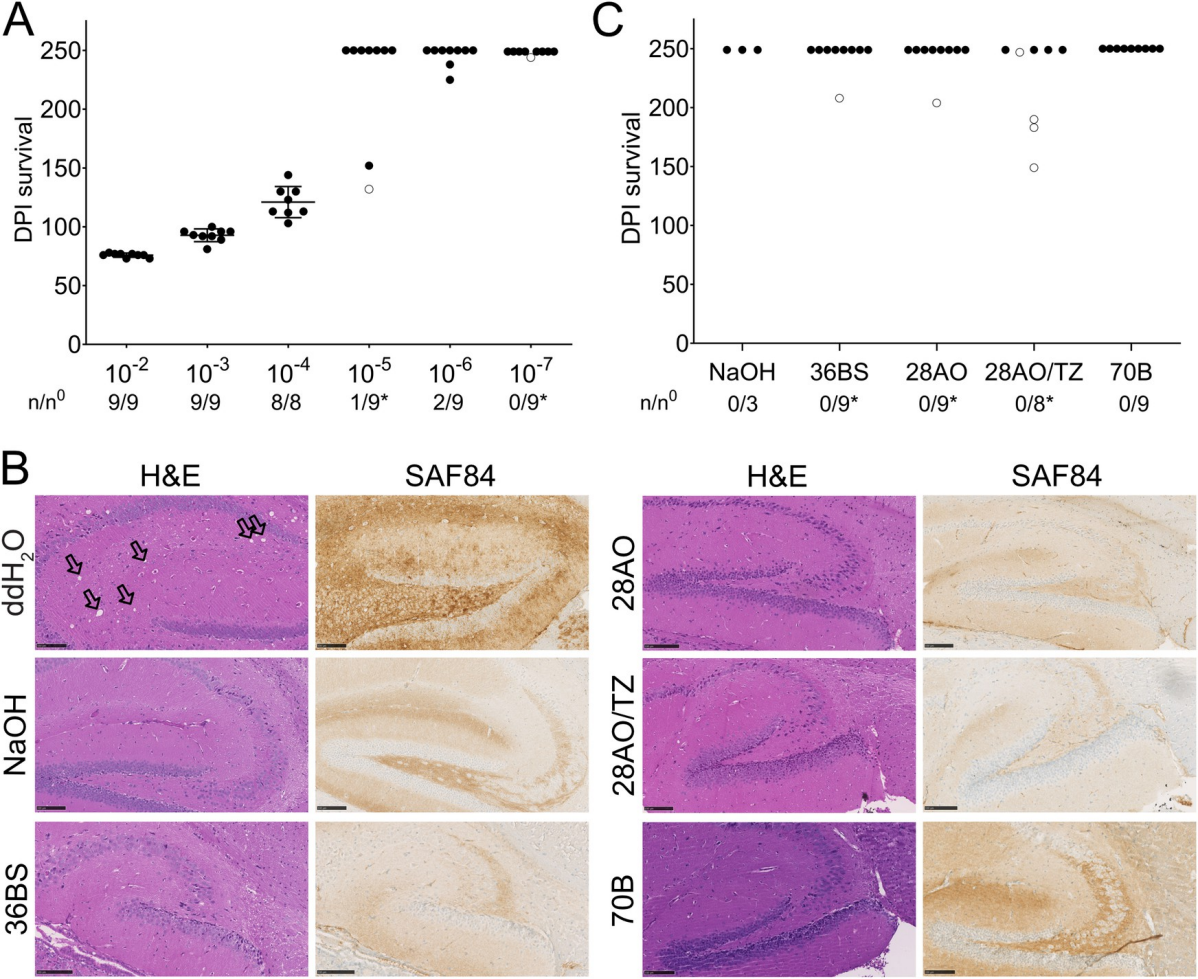
Mouse bioassay for the evaluation of the decontamination efficiency of the different formulations. **(A)** End-point titration in *tg*a*20* mice inoculated with beads exposed to 10-fold serial dilutions of RML6 from 10^−2^ to 10^−7^. Data points are shown as the mean incubation time ± standard deviation (SD) of the mean for dilutions 10^−2^ to 10^−4^. n/n^0^: indicates the attack rate (number of mice developing a prion disease divided by the total number of inoculated mice). Open circles: individual mice that died of an intercurrent death unrelated to prion infection. **(B)** Histopathology of brain sections of the hippocampus from *tg*a*20* mice inoculated with RML6-exposed beads treated with either ddH_2_O, NaOH or the different formulations (as indicated in the Figure). Vacuolation along with PrP^C^ and PrP^Sc^ deposition, serving as markers for the presence of prion disease, was observed upon visualization through H&E staining and imaging with an antibody targeting PrP (SAF84) in brain sections of mice inoculated with RML6-coated beads treated with ddH_2_O (see also S3 Fig). No vacuolation and PrP^Sc^ deposits were observed in the brains of mice inoculated with RML6-coated beads treated with NaOH or the different formulations. The SAF84 staining for 70B is more intense than for the other slides due to a stronger background signal of PrP^C^ resulting from the use of a thicker slide that was necessary as the sectioning was affected by the presence of the beads in the brain. The use of a thicker slide also causes a more intense H&E staining for 70B. Coronal sections are presented for mice inoculated with RML6-coated beads treated with ddH_2_O, while sagittal sections are shown for all other mice. Vacuoles are indicated by black arrows. (Scale bars: 100 μm). **(C)** Survival of *tg*a*20* mice inoculated with RML6-exposed beads treated either with NaOH or the different formulations. None of the mice developed a prion disease until the end of the experiment.

We then selected formulations 70B, 28AO, 28AO/TZ, and 36BS to further assess their prion decontamination effectiveness. We first analyzed prion-coated beads after treatment with the different formulations and NaOH for the presence of PrP^Sc^ by PK immunoblotting (Fig 2C). After treatment, no residual PK-resistant PrP^Sc^ was detectable on the immunoblot, showing that all decontamination products effectively reduced PK-resistant PrP^Sc^. To investigate if the identified formulations were also able to reduce prion infectivity on the prion-coated beads, we further confirmed their decontamination capability in *tg*a*20* mice, a transgenic mouse line that overexpresses murine PrP^C^ [41]. We first determined the sensitivity and maximal titer reduction that can be achieved in the bioassay after decontamination of the prion-coated beads by performing an end-point dilution titration. Three independent batches of 25 mg beads were incubated in 100 μL of 10-fold serial dilutions of RML6 (10^−2^–10^−7^; Table 3). Bead suspensions of 30 μL (750 μg beads/mouse) were then inoculated intracerebrally (i.c.) into three groups of three *tg*a*20* mice each (n = 9 per dilution, from three independent bead preparations). The survival of the inoculated mice was monitored for 250 days post infection (dpi). Mice inoculated with beads exposed to RML6 dilutions from 10^−2^ to 10^−6^ developed clinical signs of a prion disease after incubation periods between 74 and 238 dpi (Fig 3A, Table 3).

**Table 3:** Summary of the mouse bioassays to determine the decontamination efficacy of the different formulations.

End-point titration of RML6 ^a^			Steel bead *in vivo* assay			Decontamination ^c^		
RML6 ^b^ dilution	Mean incubation period (days ± SD)	Titer (Log LD_50_ units ml^-1^)	RML6 dilution to coat the beads	Mean incubation period (days ± SD)	Estimated titer (Log LD_50_ units ml^-1^)	Chemical	Incubation periods (days)	Red. (log_10_)
10^−1^	54 ± 1	7.9				NaOH	>250	≥5.1
10^−2^	59 ± 2	6.9	10^−2^	75.9 ± 1.8	5.1	36 BS	>250	≥5.1
10^−3^	69 ± 1	5.9	10^−3^	92.8 ± 5.5	3.6	28 AO	>250	≥5.1
10^−4^	77 ± 2	4.9	10^−4^	121 ± 13.2	1.2	28 AO/TZ	>250	≥5.1
10^−5^	86 ± 2	3.9	10^−5^	152, >250	-	70B	>250	≥5.1
10^−6^	93 ± 2	2.9	10^−6^	225, 238, >250	-			
10^−7^	119 ± 4	1.9	10^−7^	>250	-			
10^−8^	121, >236	0.9						
10^−9^	>236	-						

^a^ The end-point titration data for RML6 in *tga*20 mice were adapted from Falsig, Julius et al. [32]. The mean incubation periods for dilutions ranging from 10^−2^ to 10^−7^ from these data were used to calculate an approximate linear regression curve (y = -0.086x + 11.63; r = 0.94), that was used as calibration curve for the estimation of the titers of the prion-coated beads using the incubation time method [45–47].

^b^ Dilutions were started from a 10% BH.

^c^ Treatment with NaOH and the different formulations was performed on beads coated with a 10^−2^ dilution of RML6. All formulations reduced the prion infectivity titer by at least 5.1 log_10_. Red.: titer log reduction.

To further confirm that mice succumbed to prion disease, brains (10^−2^ dilution of RML6) were extracted and immunohistologically investigated for the presence of spongiform changes and the accumulation of PrP^Sc^ (Fig 3B and S3 Fig). Survival data were then analyzed by comparison to a standard curve obtained from an end-point titration of RML6 inoculated *tg*a*20* mice [32] and a median 50% lethal dose [LD_50_] of about 5.1 log_10_ infectious units ml^-1^ was estimated for the prion-exposed bead suspension inoculated per mouse (Table 3). This value represents the maximum sensitivity achievable in the mouse bioassay with the selected amount of beads and is similar to the prion titer previously determined in mice for individual steel wires (10^5.5^ LD_50_ wire units) coated with either RML [42] or the 263K hamster strain [43, 44].

We next explored the efficiency of 70B, 28AO, 28AO/TZ, and 36BS to reduce prion infectivity on the prion-coated beads in the bioassay. 30 μL of prion-exposed beads (1% (w/v) RML6) treated with the different formulations or NaOH (same samples/conditions as used in the TESSA (Fig 2A and 2B, S1 Fig) were inoculated i.c. into *tg*a*20* mice in three groups of three animals. All mice inoculated with prion-exposed beads treated with the different formulations (n = 9 per formulation) showed no symptoms of prion disease and survived for at least 250 dpi. The absence of disease was further confirmed by immunohistochemical analysis using hematoxylin and eosin (H&E) and SAF84 staining of brain sections from these mice (Table 3, Fig 3B and 3C, S3 Fig). To further investigate the absence of any seeding active prions in the brains of these mice, homogenized brain samples of three individual mice per group were analyzed by a standard RT-QuIC [28, 29]. Almost no signal was observed for any of the BH of mice previously inoculated with the treated beads, indicating the absence of seeding active prions (S4 Fig). On the contrary, samples from mice inoculated with ddH_2_O treated prion-exposed beads showed a positive response. Our data thus indicate that all formulations and NaOH reduced prion infectivity on the prion-exposed beads below detectability in the mouse bioassay. This corresponds to a titer reduction of ≥ 5.1 log_10_ units ml^-1^ based on the established reference curve for prion-exposed beads. In conclusion, our data show that all tested formulations are highly effective for the inactivation of prions on medical steel surfaces.

## Discussion

In this study, we have developed TESSA, a stainless-steel bead RT-QuIC assay, as a tool for the rapid screening of a large number of chemical formulations to assess their effectiveness to inactivate prions on steel surfaces. We chose micron scale stainless steel beads as prion carriers to mimic the surface of surgical steel instruments. These beads can be easily pipetted and are thus more practical and safer to handle compared to previously used prion carriers [9, 17, 48–50]. Additionally, we air-dried the beads after prion exposure to perform the screen under most stringent conditions, since air-dried prions bind more tightly to surfaces and exhibit higher resistance to decontamination treatments [19]. Using this approach, our results showed that the steel beads were able to efficiently bind biologically active prions in a way identical to that previously described for prions adhered to other carriers [9, 17, 49–51].

For the screen, we focused on investigating strong to mild alkaline (> pH 9.5) and oxidative formulations which we grouped in four classes based on their main anti-prion ingredients. We identified 70B, a hypochlorite-based formulation, as the most effective, while all alkaline formulations tested in the screening (categories 1–3) showed reduced efficacy under conditions simulating automated washer-disinfector cleaning processes (55°C, 10 min). Formulation 70B proved to be highly efficient at a concentration of 1% (v/v) and at even lower concentrations and shorter contact times (0.5% v/v, 5 min). Additionally, we found that the alkaline formulations, 28AO [37], 28AO/TZ, and 36BS, used for the applicability testing of TESSA, also abolished the propagation activity of the steel surface adhered prions. However, we observed a few weak false-positive reactions near our set threshold for 36BS and 28AO, as occasionally occur in RT-QuIC assays [9, 29], which we considered as unspecific reactions. The subsequent bioassay ultimately confirmed their false-positive nature.

After having identified 70B, 28AO, 28AO/TZ and 36BS as being highly efficient in TESSA, we further confirmed their effectiveness by PK immunoblotting where all formulations eliminated the amount of bead bound PrP^Sc^ below detectability. Although *in vitro* SAAs are now more widely applied for the evaluation of prion decontaminants [9, 17, 49–52], bioassays are still required to claim a new formulation as prionicidal [13, 25, 53]. In this study, all four formulations, along with the reference NaOH, reduced prion titers in the mouse bioassay below the detection limit of the maximum achievable sensitivity of 5.1 LD_50_ ml^-1^ which was determined in the reference bioassay. These results demonstrate that all formulations fulfilled or even exceeded the criteria of a log reduction of 4.5 in infectivity, required by the WHO for a prion decontaminant to be considered as effective [20]. The data for 28AO also confirm previous *in vitro* experiments [37], and thus provide further evidence for its prionicidal efficacy.

In line with previous studies [9, 29, 54–58], our results reinforce the close correlation between data obtained by RT-QuIC assays and mouse bioassays, supporting the use of modified RT-QuIC assays, such as TESSA, as viable methods for the screening and preselection of new prion decontaminants. However, an inherent limitation persists in the use of these assays for evaluating the efficacy of novel decontaminants [9]. These assays seem to exhibit greater sensitivity than rodent bioassays, which may result in false-positive reactions and consequently to the exclusion of effective decontamination products from further investigation. While this may not necessarily raise medical safety concerns, obtaining definitive answers on whether SAAs can serve as a complete substitute for the biological infectivity determined by bioassays still requires additional data for a final conclusive assessment.

An important prerequisite for effective prion decontaminants is not only that they efficiently eliminate any prion contamination, but also that they are compatible with corrosion-sensitive materials to avoid damage to delicate surfaces of medical devices. Formulations 28AO, 28AO/TZ, and 36BS are gentle alkaline cleaners which are applicable to corrosion-sensitive surfaces of medical devices. This property, along with our finding for effective prion inactivation, suggests their potential application as an additional safety measure in the multistep reprocessing procedures of reusable medical devices applied in medical settings as advised by the WHO [20] to increase the decontamination efficiency and reduce the risk of iCJD transmission through unintentionally contaminated surgical instruments.

Chlorine-based disinfectants, such as sodium hypochlorite, belong to the most effective prion decontaminants [20, 49, 59], but have limited relevance for routine instrument reprocessing due to their strong corrosive properties. In our work, formulation 70B, designed to have a low active chlorine concentration to meet the requirement for low corrosion properties, turned out to be a highly effective decontaminant that reduced the prion infectivity titer below detectability. However, chlorine-based products are constrained by their limited shelf life due to degradation of the active chlorine content over time [9, 39, 40]. The simultaneous decline in the active chlorine content and decontamination proficiency was also observable in our findings with formulation 70B. We therefore advise for using either freshly prepared solutions or the addition of chlorine stabilizers. Under these conditions, formulation 70B could be a useful alternative cleaner for the decontamination of prion contaminated surfaces.

The effectiveness of prion decontaminants can also depend on the prion strain type [12, 25, 42, 44, 48, 60, 61]. Due to the scarcity of suitable human prion mouse models [62, 63], we relied on the rodent-adapted prion strain, RML6, which has been widely used as a model strain for the development and validation of prion decontaminants (Fichet, Comoy et al. 2004, Lemmer, Mielke et al. 2008, Edgeworth, Sicilia et al. 2011, McDonnell, Dehen et al. 2013). However, the efficacy of prion inactivation procedures tested on rodent prions cannot be completely generalized to human prions [54, 64, 65]. In the future, however, SAAs such as TESSA, that are applicable to many different prion strains [9, 66, 67], could be used as animal-free methods for the additional validation of prion decontaminants on multiple prion strains.

In conclusion, TESSA allows the detection of steel-bead adsorbed prions and represents an effective method for the rapid screening and evaluation of the effectiveness of new prion decontaminants on steel surfaces. Our data further show that formulations 28AO, 28AO/TZ, 36BS and 70B efficiently inactivate prions below the detection limit as demonstrated by several orthogonal *in vitro* methods as well as by a mouse bioassay. These formulations could therefore be suitable as mild yet effective, anti-prion decontaminants for routine instrument reprocessing to increase the safety of reusable surgical and medical instruments.

## Materials and methods

### Chemical compositions of the formulations

Products and conditions used in this study and summarized in Tables 1 and 2 were formulated and provided by Borer Chemie AG (Zuchwil, Switzerland). During the TESSA screening experiments, experimenters were blinded for the content of the different formulations and results were provided to Borer Chemie AG. TESSA results were used in a positive feedback loop to further improve the anti-prion effectiveness of the formulations. Four different main chemical categories were tested, including series of mild alkaline alkanolamine (Category 1), alkaline enzymatic phosphate/silicate (Dec TWIN PH10; Category 2), strong alkaline hydroxide- and silicate-based (deconex^®^ OP 152, deconex^®^ HT and deconex^®^ MT; Category 3), and hypochlorite-based formulations and solutions (Category 4). In the product lines deconex^®^ OP 152, deconex^®^ HT and deconex^®^ MT, OP denotes cleaning products used in optics and refers to high-performance products for residue-free cleaning in the production of ophthalmic lenses and precision optics; MT refers to products used in medicine technology, that are cleaning agents based on the high requirements of medical device manufacturers for residue-free cleaning in the production of Medtech products and HT historically stands as an abbreviation for high-tech, pertaining to cleaning agents applied in industry for residue-free cleaning in production. To improve the anti-prion and cleaning properties of the formulations in category 1 and 2, they were supplemented with different ingredients. Enzymatic detergents, such as amylases, cellulases and lipases, were added for the digestion and removal of lipids, carbohydrates, polysaccharides and fatty deposits [68], whereas proteases were used to hydrolyze and remove proteins and to directly act on prions [69]. In addition, non-ionic surfactants, chelating agents and surface active reagents (e.g. fatty alcohol alcoxylates and alkyl glucosides) were supplemented to induce a low-foam profile that is required for automated applications, and/or as corrosion inhibitors or wetting reagents to reduce the surface tension [70]. In addition, the raw product and components (S1 Table) were tested for 70B.

### Coating of the stainless-steel beads with prions and decontamination procedure

Unless otherwise specified, 100 μL of 1% RML6 brain homogenate (in-house produced, 9.9 ± 0.2 log LD_50_ units g^-1^ [32]) or NBH were incubated with 25 mg of autoclaved stainless steel beads (AISI 316L –grade surgical steel, average diameter of <20 μm; Thyssenkrupp; material number 1.4404) for 2 h at 37°C under agitation at 1100 rpm. After incubation, the supernatant was discarded, and beads were air-dried at 37°C for 1 h to strengthen the adherence of the prions to the beads. Beads were washed four-times with sterile phosphate-buffered saline (PBS) to remove any residual RML6 using a DynaMag-2 (Thermo Fisher) for magnetic separation of the beads from the buffer and stored at -20°C until further use. For prion-inactivation, RML6-coated beads were treated with the different formulations as specified in Tables 1 and 2 under constant shaking at 1’100 rpm. As positive or negative controls, RML6-coated beads were treated with either 1M NaOH or ddH_2_O, respectively, under agitation at 1100 rpm for 2 h at room temperature (RT). After the treatment, beads were washed 10 times with ddH_2_O and stored in 1 mL PBS at -20°C until further usage.

### TESSA

For the TESSA reactions, the same experimental conditions were used as previously described for a standard RT-QuIC [28, 31, 71]. Briefly, the reaction buffer of TESSA was composed of recombinant hamster PrP(23–231) (produced in-house; filtered using 100 kDa centrifugal filters (Pall Nanosep OD100C34)) at a final concentration of 0.1 mg/mL, 1 mM EDTA, 10 μM Thioflavin T, 170 mM NaCl and PBS (incl. 130 mM NaCl) [28]. To the 70 μL reaction buffer per well (96-well plate), RML6- coated beads either untreated or treated with the different formulations as described above were added (30 μL of 25 mg/mL ~ 750 μg). The TESSA reactions were performed in a FLUOstar Omega plate reader (BMG Labtech) with cyclic shaking modes of 7 × (90 s shaking (900 rpm (double orbital), 30 s rest)), 60 s reading) at 42°C. Reading was carried out with excitation at 450 nm and emission at 480 nm with a gain of 2000 every 15 min for 105 h. For each run, reactions were performed in quadruplicates. Each microplate comprised four control wells containing RML6-coated beads either treated with ddH_2_O or 1 M NaOH as positive and negative controls, respectively. Only fluorescence positive reactions between 0 and 83 h (~5’000 min) were considered for the data evaluation, because of an increasing occurrence of spontaneous aggregation events at later reaction times. To further exclude fluctuations from the baseline, a threshold was set arbitrarily at 30’000 RFU which corresponds to ~200% [29, 72] of the mean fluorescence reading for the negative control measurements assessed during the establishment of TESSA.

### Immunoblotting

Samples containing beads were prepared following the procedure as mentioned above. As controls, NBH or RML6 were used after determination of their total protein levels with a bicinchoninic acid assay (BCA, Pierce) according to the manufacturer’s instruction. To assess PK-resistant PrP^Sc^ levels on the beads, the protein levels of the homogenates were adjusted to 20 μg of total protein. Samples were then subjected to PK digestion. Briefly, 20 μL PBS with PK (25 μg/mL) was added to the beads. The PK digestion was performed directly on the beads at 37°C for 30 minutes with continuous shaking at 1’100 rpm. To stop the digestion and to release the proteins from the beads, 6 μL of 4 × LDS containing loading dye (NuPAGE, Thermo Fisher) and 1 mM 1,4-dithiothreitol (Roche) were added prior to boiling the samples at 95°C for 10 minutes and centrifugation at 2’000 rpm for 5 minutes. The protein-containing supernatants were loaded on a 12% Bis-Tris Gel (NuPAGE, Thermo Fisher) and subsequently transferred to a nitrocellulose membrane using the iBlot system (Thermo Fisher). Membranes were blocked with 5% SureBlock (LuBio science) and probed with 1:10’000 monoclonal in-house produced POM1 antibody [36] in 1% Sure-Block/PBS with 0.1% Tween-20 (PBS-T). As a detection antibody, an HRP-conjugated goat anti-mouse antibody (1:10’000, Bio-Rad Laboratories) was used in 1% SureBlock-PBS-T. Membranes were developed with Crescendo HRP substrate (Millipore) and imaging was done using the LAC3000 system (Fuji).

### Mouse bioassay in *tga*20 mice

All animal experimentation was performed under compliance with the rules and regulations by the Swiss Confederation on the Protection of Animal Rights. All protocols used in this study were approved by the Animal Welfare Committee of the Canton of Zurich under permit number ZH040/15. *Tga20* mice were kept under general anesthesia after isoflurane treatment and inoculated i.c. using 30 μL of a suspension of RML6-coated beads that were either untreated or treated with the different decontaminants as described above. Inoculated mice were subjected to health monitoring every second day until the appearance of terminal clinical symptoms of scrapie. On the first day the animals presented themselves as being terminally sick, they were sacrificed under isoflurane anesthesia. Brains of mice were dissected and analyzed immunohistochemically for the presence of a prion disease. Histological analysis was performed on one hemisphere of the dissected brains after inactivation in 96% formic acid and fixation in formalin. Sections of 2 μm thickness, were then cut onto positively charged silanized glass slides. In the case of mice inoculated with RML6-coated beads treated with 70B, sections with a thickness of 5 μm were utilized due to the influence of the presence of the beads, which affected the cutting process. The brains of mice inoculated with RML6-coated beads treated with ddH_2_O were cut into coronal sections, while the brains of all other mice were sectioned sagittally. Sections were then either stained with H&E to confirm spongiform changes or subjected to immunostaining using a PrP-specific antibody (SAF84; SPI bio; 1:200 for 32 minutes) to visualize PrP^Sc^ aggregates as reported previously [47]. Immunohistochemical staining was performed using an automated NEXES immunohistochemistry staining apparatus (Ventana Medical Systems, Switzerland) with an IVIEWDAB Detection Kit (Ventana).

BHs from tga20 mice inoculated with the beads were also analysed by RT-QuIC for the presence of propagation active prions. Brains were homogenized in 0.32 M sucrose (10% (w/v), Sigma) using the Precellys 24 (Bertin Instruments) as previously described [28] and diluted 20’000 times in PBS. A total of 98 μL of reaction buffer was dispensed into a well of a 96-well plate and 2 μL of each BH sample was added. The standard RT-QuIC assay was then performed under the conditions as described above. RML6 and NBH were used as positive and negative controls, respectively.

### Titer determination

Titers of RML6-coated beads were determined by the incubation time method as previously described [45–47]. Briefly, beads exposed to 10-fold serial dilutions (9.9 ± 0.2 log LD_50_ units g^-1^) ranging from 10^−2^ to 10^−7^ were injected i.c. into *tga*20 mice. At the terminal stage of the disease, the incubation times were correlated to an approximate linear regression curve obtained from the previously determined mean incubation periods of mice inoculated with serial 10-fold dilutions of RML6 [32]. By comparing the incubation times to this calibration curve, a titer of ~ 5.1 log_10_ LD_50_ units ml^-1^ was obtained for beads coated with a 10^−2^ dilution of RML6. For the efficacy testing of the different formulations, beads coated with 10^−2^ dilutions of RML6 (~ 5.1 log_10_ LD_50_ units ml^-1^) were used.

## Supporting information

S1 Fig Additional TESSA replicates to confirm the decontamination efficacy of 28AO, 28AO/TZ and 36BS. Same as shown in Fig 2A, but for three further TESSA replicates of RML6-exposed beads after decontamination with formulation 28AO, 28AO/TZ and 36BS. Shown are three TESSAs for each condition in quadruplicates. The dashed line indicates the ThT fluorescence threshold for a positive reaction.
(TIF)

S2 Fig TESSA controls and further replicates to confirm the decontamination efficacy of 70B. **(A)** Additional representative controls used in TESSA. RML6-exposed beads treated with either ddH_2_O or NaOH were used as positive and negative controls, respectively, on each microplate. **(B)** Same as shown in Fig 2B, but for three further replicates of RML6-exposed beads after decontamination with formulation 70B.
(TIF)

S3 Fig Additional histopathological brain images. Same as in Fig 3B, but whole mount brain slices (left two panels) and brain sections depicted at lower magnification (right two panels) are shown to better visualize the typical PrP^Sc^ deposits in the brain slides of *tga*20 mice inoculated with RML6-coated beads treated with ddH_2_O (ddH_2_O) and their absence in the slides of mice inoculated with prion-coated beads treated with either NaOH or the different formulations (Scale bars left two panels: 2.5 mm, right two panels: 250 μm).
(TIF)

S4 Fig Standard RT-QuIC analysis of BHs from *tg*a*20* mice inoculated with RML6-coated beads treated with ddH_2_O or the different decontaminants. Standard RT-QuIC analysis of BHs of three individual mice per condition in quadruplicates.
(TIF)

S1 Raw images Uncropped immunoblots. **(A)** Uncropped immunoblot of Fig 1A. Left panel: immunoblot stained with POM1, right panel: marker. **(B)** Uncropped immunoblot of Fig 2C. Left panel: immunoblot stained with POM1, right panel: marker.
(PDF)

S1 Table TESSA of RML6-exposed beads treated with the individual ingredients of 70B and of NBH-coated beads treated with 70B. All reagents and 70B were applied at 55°C for 10 min.
(DOCX)

S1 Dataset Raw data for TESSA and RT-QuIC reactions. (XLSX)
