# Supplementary figures and images for: Advancing surgical instrument safety: A screen of oxidative and alkaline prion decontaminants using real-time quaking-induced conversion with prion-coated steel beads as surgical instrument mimetic
Source: PLoS One. 2024 Jun 13;19(6):e0304603. doi: 10.1371/journal.pone.0304603 (PMC11175539; doi:10.1371/journal.pone.0304603)

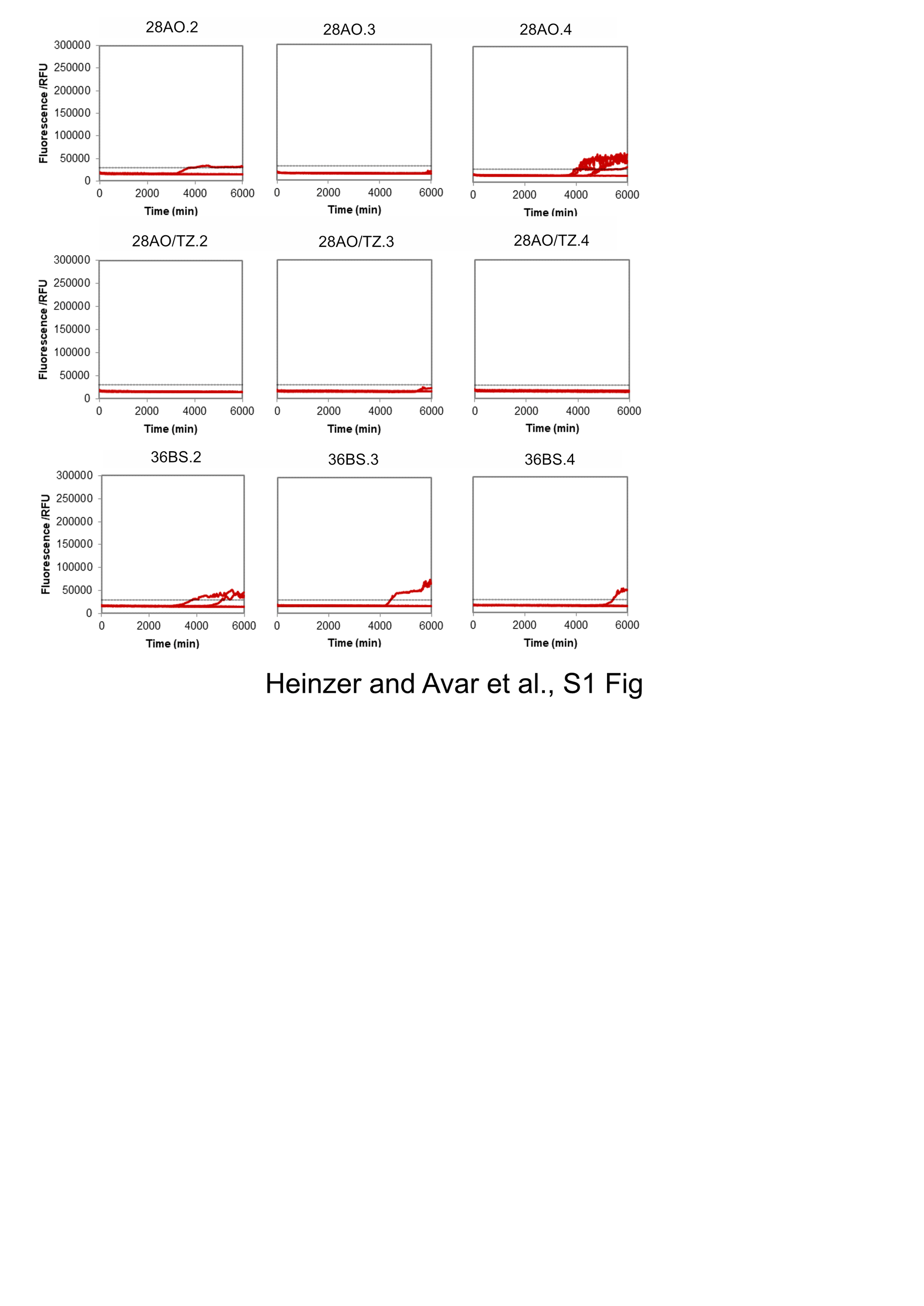

Supplement: S1 Fig — Same as shown in Fig 2A, but for three further TESSA replicates of RML6-exposed beads after decontamination with formulation 28AO, 28AO/TZ and 36BS. Shown are three TESSAs for each condition in quadruplicates. The dashed line indicates the ThT fluorescence threshold for a positive reaction. (TIF) [file pone.0304603.s001.tif]

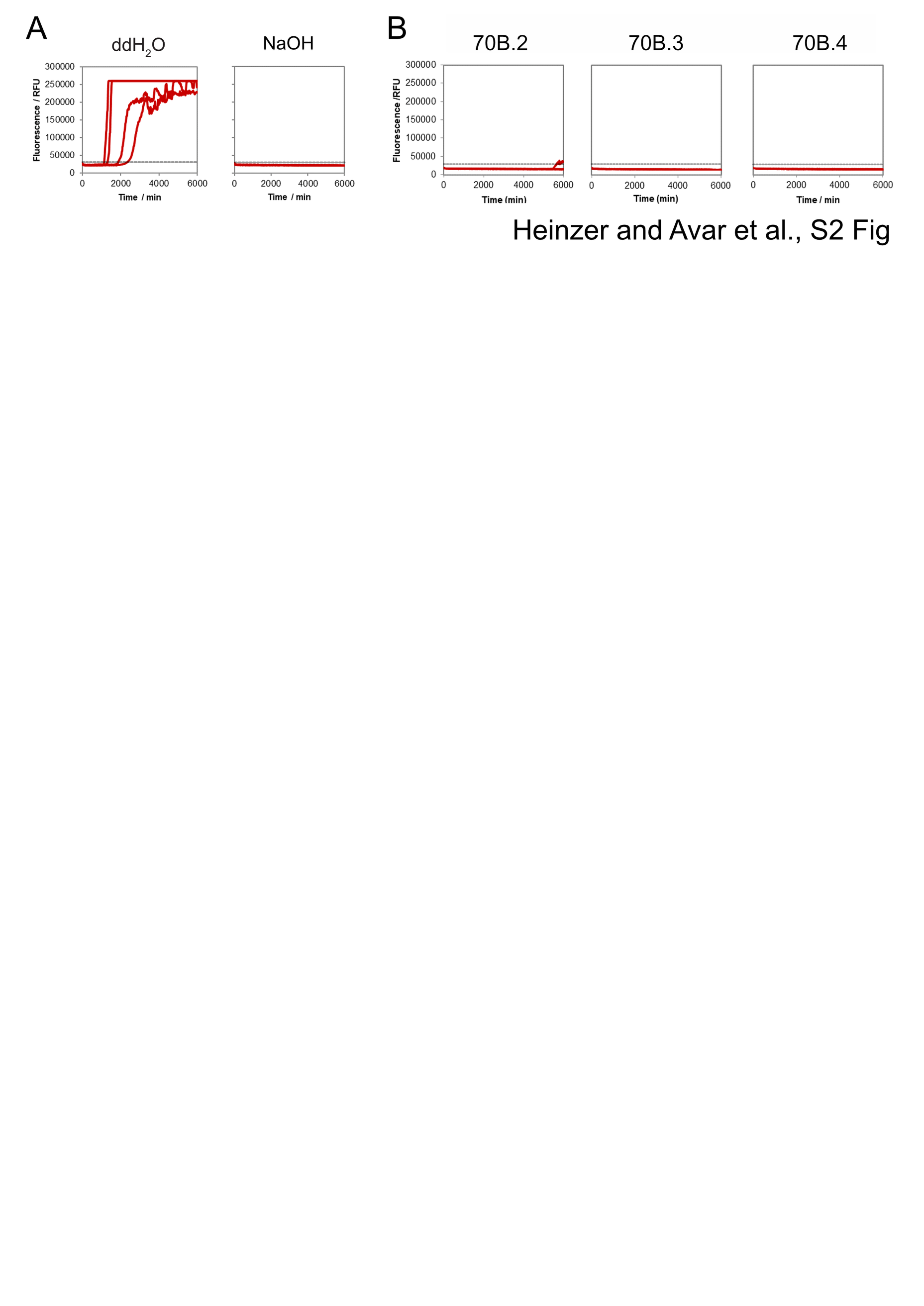

Supplement: S2 Fig — (A) Additional representative controls used in TESSA. RML6-exposed beads treated with either ddH2O or NaOH were used as positive and negative controls, respectively, on each microplate. (B) Same as shown in Fig 2B, but for three further replicates of RML6-exposed beads after decontamination with formulation 70B. (TIF) [file pone.0304603.s002.tif]

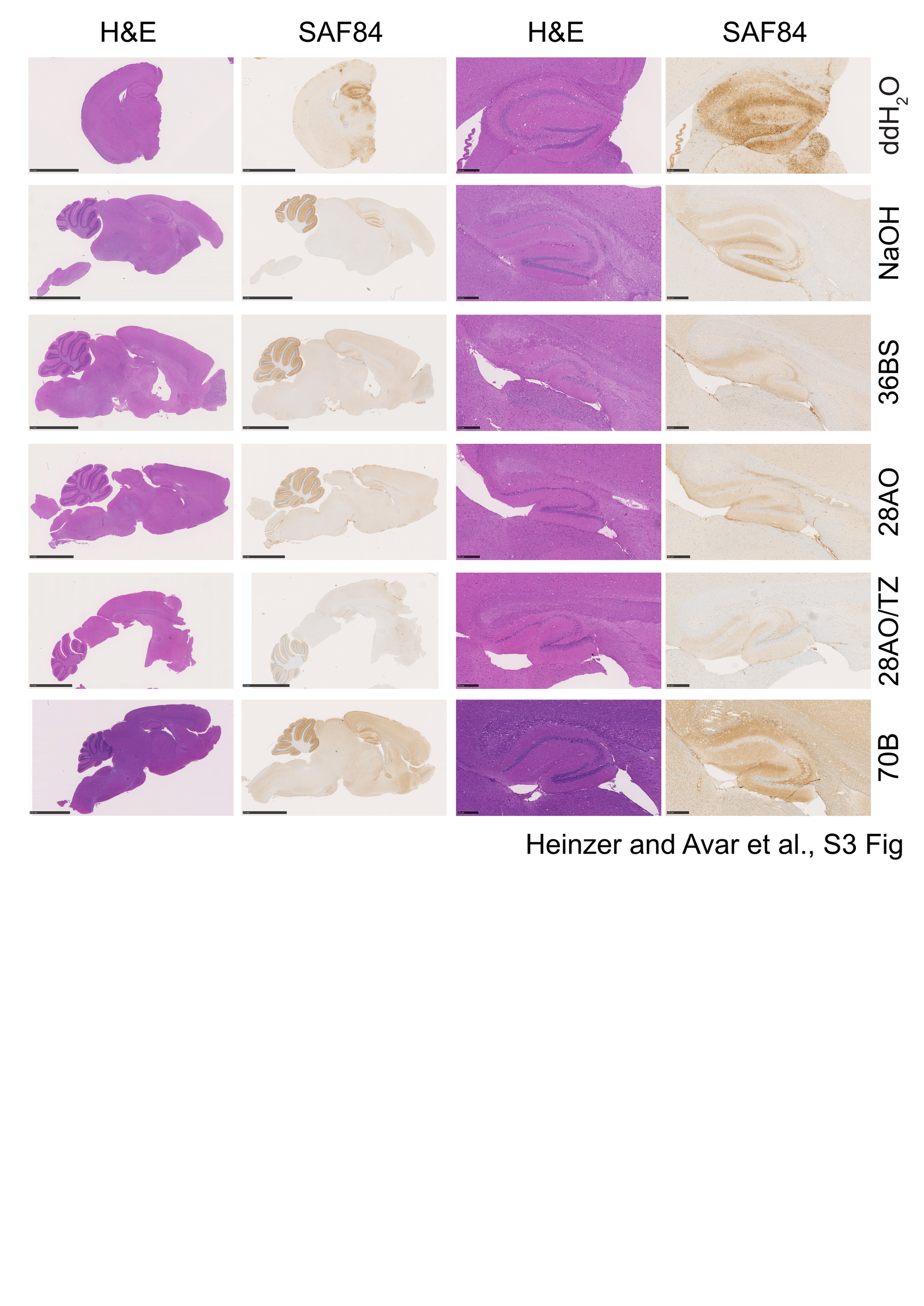

Supplement: S3 Fig — Same as in Fig 3B, but whole mount brain slices (left two panels) and brain sections depicted at lower magnification (right two panels) are shown to better visualize the typical PrPSc deposits in the brain slides of tga20 mice inoculated with RML6-coated beads treated with ddH2O (ddH2O) and their absence in the slides of mice inoculated with prion-coated beads treated with either NaOH or the different formulations (Scale bars left two panels: 2.5 mm, right two panels: 250 μm). (TIF) [file pone.0304603.s003.tif]

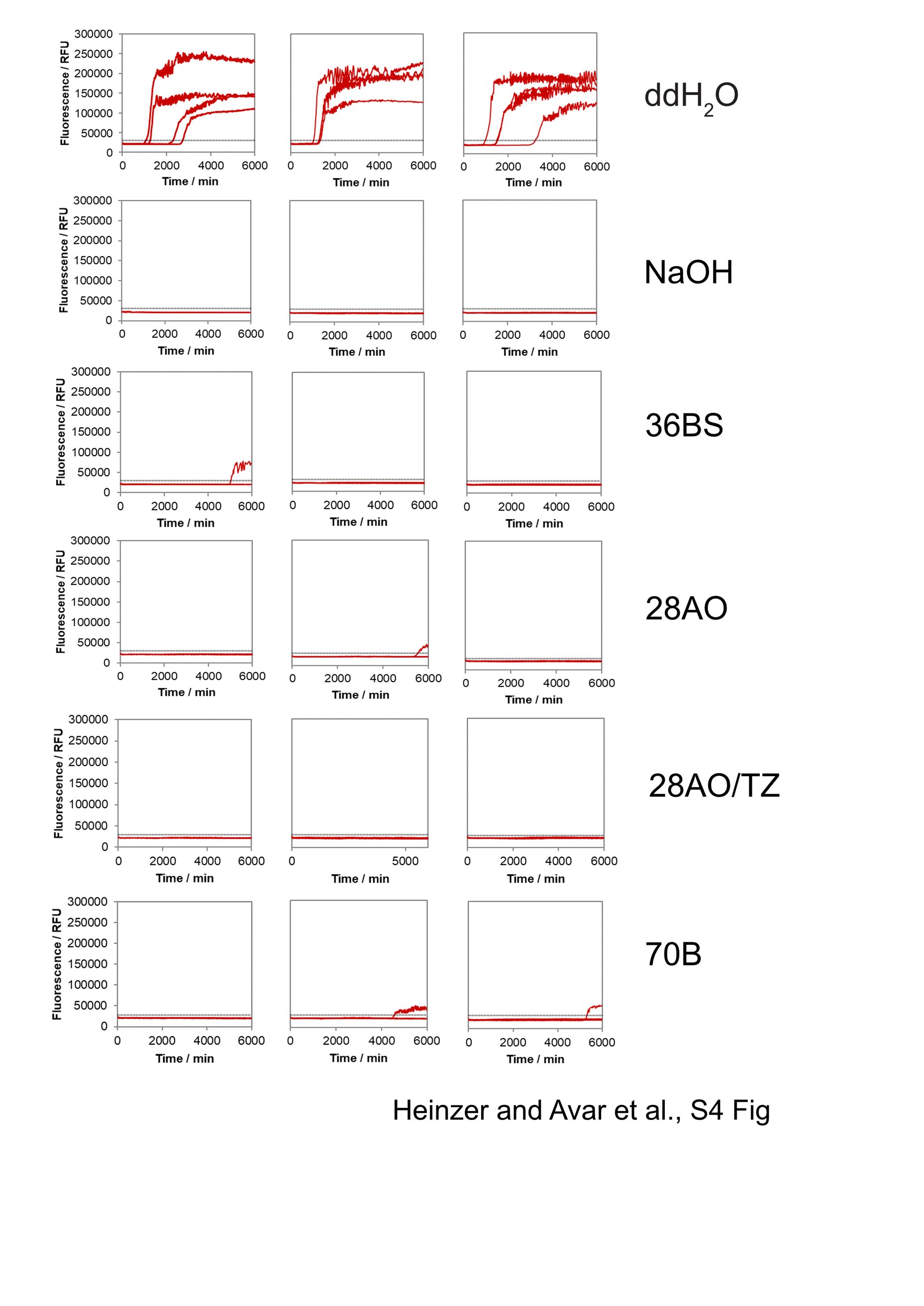

Supplement: S4 Fig — Standard RT-QuIC analysis of BHs of three individual mice per condition in quadruplicates. (TIF) [file pone.0304603.s004.tif]
