# Supplementary material for: Advancing surgical instrument safety: A screen of oxidative and alkaline prion decontaminants using real-time quaking-induced conversion with prion-coated steel beads as surgical instrument mimetic
Source: PLoS One. 2024 Jun 13;19(6):e0304603. doi: 10.1371/journal.pone.0304603 (PMC11175539; doi:10.1371/journal.pone.0304603)
Supplement: S1 Raw images — (A) Uncropped immunoblot of Fig 1A. Left panel: immunoblot stained with POM1, right panel: marker. (B) Uncropped immunoblot of Fig 2C. Left panel: immunoblot stained with POM1, right panel: marker. (PDF) [file pone.0304603.s005.pdf]

Western Blot to Figure 1A

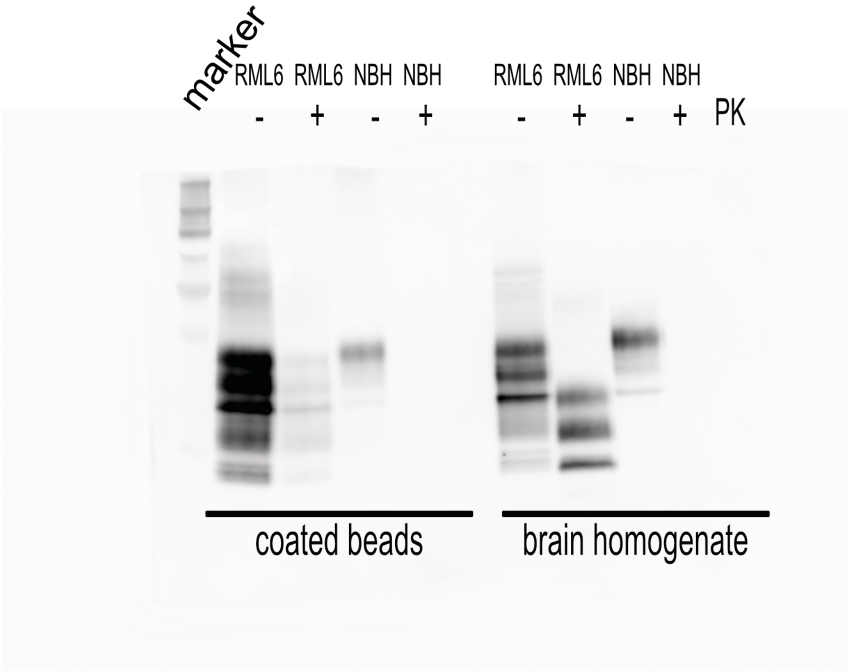

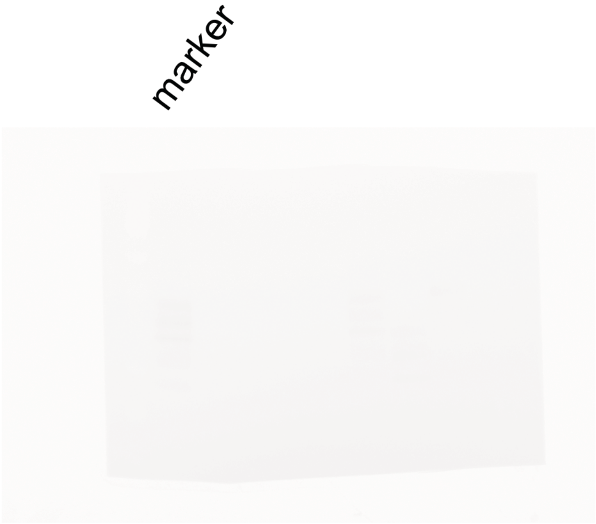

Western Blot to Figure 2C

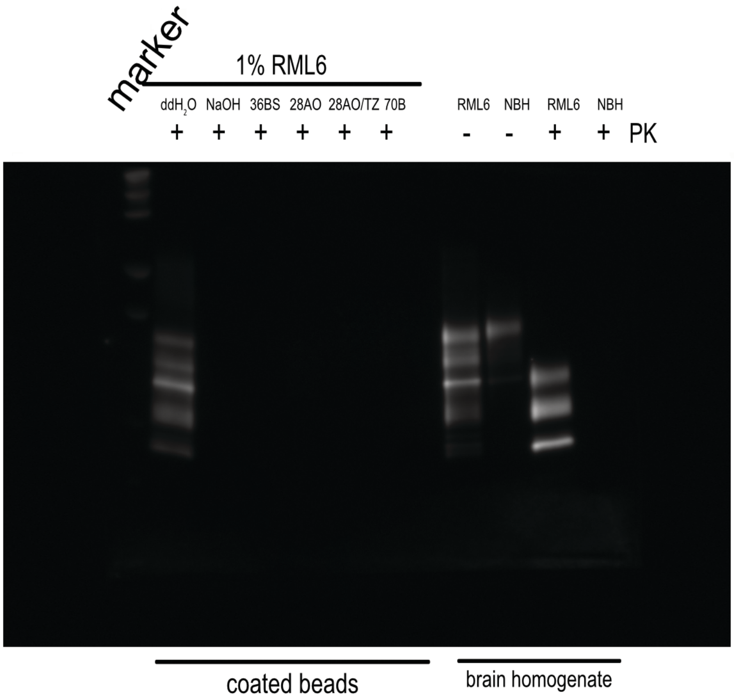

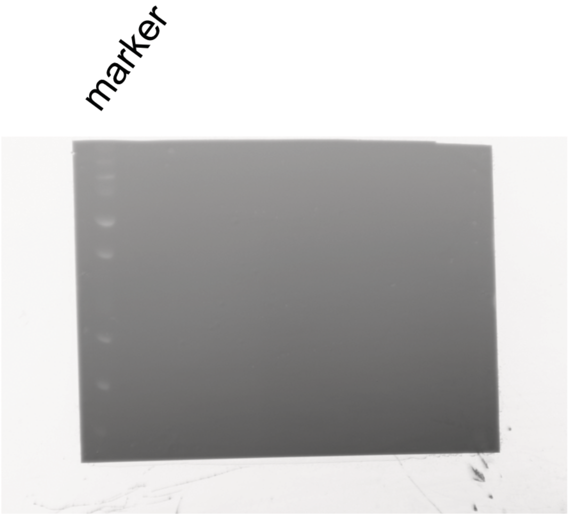

Membranes were developed with Crescendo HRP substrate (Millipore) and imaging was done using the LAC3000 system (Fuji).
