# Supplementary material for: Advancing surgical instrument safety: A screen of oxidative and alkaline prion decontaminants using real-time quaking-induced conversion with prion-coated steel beads as surgical instrument mimetic
Source: PLoS One. 2024 Jun 13;19(6):e0304603. doi: 10.1371/journal.pone.0304603 (PMC11175539; doi:10.1371/journal.pone.0304603)
Supplement: S1 Table — All reagents and 70B were applied at 55°C for 10 min. (DOCX) [file pone.0304603.s006.docx]

**S1 Table. TESSA of RML6-exposed beads treated with the individual ingredients of 70B and of NBH-coated beads treated with 70B.** All reagents and 70B were applied at 55 °C for 10 min.

| Formulation | Composition (v/v), condition | TESSA reactions  (positive/total) |
| --- | --- | --- |
| F108  F109  F110  NBH.1  NBH.2 | 1% sequestering agents, phosphonates  (raw material from 70B without hypochlorite)  1% dipotassium trioxosilicate (5-15%)  (single component of 70B without hypochlorite)  1% tetrapotassium pyrophosphate (15-30%)  (single component of 70B without hypochlorite)  1% 70B, freshly prepared  2% 70B, freshly prepared | 8/8  8/8  8/8  0/4  0/4 |
